# Implication of next-generation sequencing on association studies

**DOI:** 10.1186/1471-2164-12-322

**Published:** 2011-06-17

**Authors:** Hoicheong Siu, Yun Zhu, Li Jin, Momiao Xiong

**Affiliations:** 1 MOE Key Laboratory of Contemporary Anthropology, School of Life Sciences, Fudan University, Shanghai, 200433, China; 2 Human Genetics Center, The University of Texas, School of Public Health, Houston, TX 77030, USA

## Abstract

**Background:**

Next-generation sequencing technologies can effectively detect the entire spectrum of genomic variation and provide a powerful tool for systematic exploration of the universe of common, low frequency and rare variants in the entire genome. However, the current paradigm for genome-wide association studies (GWAS) is to catalogue and genotype common variants (5% < MAF). The methods and study design for testing the association of low frequency (0.5% < MAF ≤ 5%) and rare variation (MAF ≤ 0.5%) have not been thoroughly investigated. The 1000 Genomes Project represents one such endeavour to characterize the human genetic variation pattern at the MAF = 1% level as a foundation for association studies. In this report, we explore different strategies and study designs for the near future GWAS in the post-era, based on both low coverage pilot data and exon pilot data in 1000 Genomes Project.

**Results:**

We investigated the linkage disequilibrium (LD) pattern among common and low frequency SNPs and its implication for association studies. We found that the LD between low frequency alleles and low frequency alleles, and low frequency alleles and common alleles are much weaker than the LD between common and common alleles. We examined various tagging designs with and without statistical imputation approaches and compare their power against de novo resequencing in mapping causal variants under various disease models. We used the low coverage pilot data which contain ~14 M SNPs as a hypothetical genotype-array platform (Pilot 14 M) to interrogate its impact on the selection of tag SNPs, mapping coverage and power of association tests. We found that even after imputation we still observed 45.4% of low frequency SNPs which were untaggable and only 67.7% of the low frequency variation was covered by the Pilot 14 M array.

**Conclusions:**

This suggested GWAS based on SNP arrays would be ill-suited for association studies of low frequency variation.

## Background

Next-generation DNA sequencing platforms can effectively detect the entire spectrum of genomic variation and provide a powerful tool for systematic exploration of the universe of variants and interactions in the entire genome, and hence largely improve our ability to explore the remaining genetic variance which has not been identified by GWAS [1]. However, our knowledge of genetic variation is mainly limited to common DNA variants (i.e. minor allele frequency, 5% < MAF) and the current genetic studies of complex diseases have focused on testing associations of common alleles with common diseases. Since low frequency variants (0.5% < MAF ≤ 5%) have their inherent features that are largely different from the common genetic variants, the current strategies for association studies are well developed for identifying the association of common variants with common diseases, but may be ill-suited for large amounts of allelic heterogeneity present in sequence data [2]. To test for association of both common and low frequency alleles with the disease and the task of moving from confirmed association signal to complete enumeration of the pattern of causal variants at a given locus presents great challenges [3]. To meet these challenges, two approaches to the next wave of GWAS have been proposed [4]. One approach is to extend the current paradigm for GWAS which catalogues common variants and genotype them using chips to including low frequency variants. Companies such as Illumina and Affymetrix have already designed chips including both common and low frequency variants discovered in the 1000 Genomes Project [5]. The next wave of GWAS is to use these chips for genotyping millions of common and low frequency variants. Another approach is to conduct whole-genome and whole-exome sequencing of individuals instead of genotyping a catalogue of variants to capture both common and rare variants. To provide information for next generation GWAS which will test for association of the entire allele frequency spectrum of genomic variation with the diseases, we use the1000 Genomes Project data, including both the low coverage data (i.e. low coverage pilot) and exome capture-sequencing data (i.e. exon pilot), to study the pattern of linkage disequilibrium (LD) between common and common, common and low frequency, low frequency and low frequency variants, to assess the coverage performance of tagging designs with and without statistical imputation approaches and evaluate the power of the current widely used methods for association studies under different disease models and study designs. To evaluate the performance of DNA chips for next generation GWAS, we use the low coverage pilot data which contain ~14 M SNPs as a hypothetical genotype-array platform (Pilot 14 M) to interrogate its impact on selection of tag SNPs, mapping coverage and power of association tests.

## Results

### Allele Frequency Spectrum and Linkage Disequilibrium Pattern

We first analyze the low coverage pilot with whole genome sequencing of 179 individuals from four populations and the exon pilot with exon-targeted sequencing of 697 individuals from seven populations in the 1000 Genomes Project to examine the allele frequency spectrum and linkage disequilibrium (LD) pattern in humans.

**Figure 1:**
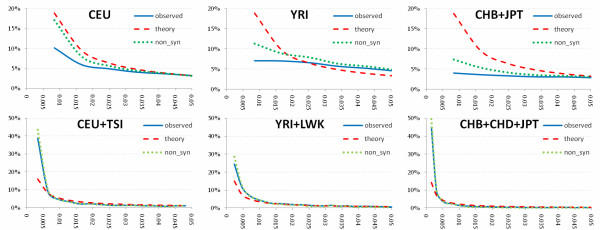
**Allele frequency distribution for low coverage pilot and exon pilot autosomal SNPs**. Figure 1 shows the MAF distribution from 0.5% to 5%. The red dashed line shows the MAF distribution expected for the standard neutral population model with constant population size and random mating, the blue solid line shows the MAF distribution for the all observed variants, and the green dotted line shows the MAF distribution for the observed non-synonymous alleles.

We observed that the proportion of the low frequency SNPs in the exon pilot dataset was much larger than that in the low coverage pilot dataset, which implied that when the number of typed individual's increases, the proportion of the low frequency SNPs dramatically increases (Additional file 1, Figure S1). This observation is consistent with the early report of Hedges et al. (2009) [6]. We can expect that when thousands of individuals are studied, the majority of SNPs will have low frequency. Figure 1 further plotted allele frequency distribution for low coverage pilot and exon pilot autosomal SNPs in CEU, YRI, CHB+JPT, CEU+TSI, YRI+LWK, and CHB+CHD+JPT, respectively. Consistent with previous studies [7], the observed frequency distribution of common alleles were much closer to the expected for the standard neutral population model, however, the proportion of SNPs with low frequency alleles is smaller than the expected for the standard neutral population model in all three populations. We observed fewer low frequency variants in CEU and CHB+JPT samples than that in YRI samples (Additional file 2, Table S1). We also observed that the distribution of allele frequency for the non-synonymous SNPs in CEU coincides well with the expected distribution of allele frequency for the standard neutral population model. Using the exon pilot data (697 individuals) the proportion of SNPs with the low frequency alleles will also increase (Figure 1). We noted the allele frequency distributions for all observed variants and non-synonymous variants in the exon pilot dataset were almost indistinguishable.

**Figure 2:**
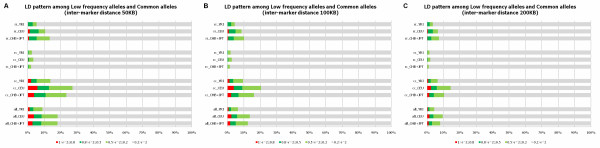
**LD pattern among low frequency alleles and common alleles**. The proportions of pair-wise SNPs with *r*^2 ^between common and common SNPs (cc), low frequency and common SNPs (rc), and low frequency and low frequency SNPs (rr) in five intervals of *r*^2 ^within each category of the MAF of SNPs (i.e., cc, rc and rr categories) for low coverage pilot dataset. We plotted graphs under three inter-marker distances, A) 50 kb, B) 100 kb and C) 200 kb where *r*^2 ^between the target SNP and its all nearby SNPs within the distance was calculated.

LD, the nonrandom association of alleles at different loci provides insight into evolutionary history of populations and lays the basis for association studies of complex diseases [8,9]. The LD between common SNPs has been well studied; there are few genome-wide surveys of LD between common and low frequency SNPs, and between low frequency and low frequency SNPs. Large-scale surveys of genome-wide LD patterns using data generated in 1000 Genomes Project will reveal the full complexity of empirical patterns of LD and facilitate research in evolutionary biology and association studies of complex diseases. A simplified view of LD is the squared correlation coefficient *r*^2 ^between the two SNPs which will be used to measure the levels of pair-wise LD in this report. If *r*^2 ^between two SNPs is larger than or equal to 0.8, then the LD between two SNPs is viewed as strong. We used inter-marker distance of 50 kb, 100 kb and 200 kb to calculate LD between SNPs. The proportions of pair-wise SNPs with *r*^2 ^in five intervals for the inter-marker distance of 50 kb, 100 kb and 200 kb based on a low coverage pilot dataset and HapMap phase II (r22) dataset are shown in Figure 2, and Additional file 3, Figure S2 respectively. Several remarkable features emerge from these figures. First, the LD patterns between common and common SNPs in the low coverage pilot dataset was consistent with that for the HapMap II, but the LD levels between common SNPs in the low coverage pilot dataset were smaller than that in the HapMap II dataset. This may be due to the fact that the distribution of the MAF in the low coverage pilot dataset was shifted to the left toward the low frequencies compared to the distribution of MAF in the HapMap II dataset. Second, the LD between common and common SNPs is much stronger than that between low frequency and low frequency SNPs, and low frequency and common SNPs. Third, in general, we observed only less than 10% of the pair-wise low frequency SNPs have strong LD. Fourth, we observed less than 2% of the pairs of low frequency and common SNPs with *r*^2 ^larger than 0.2. This demonstrated that it is difficult to use common SNPs for indirectly testing for association of low frequency variants.

**Figure 3:**
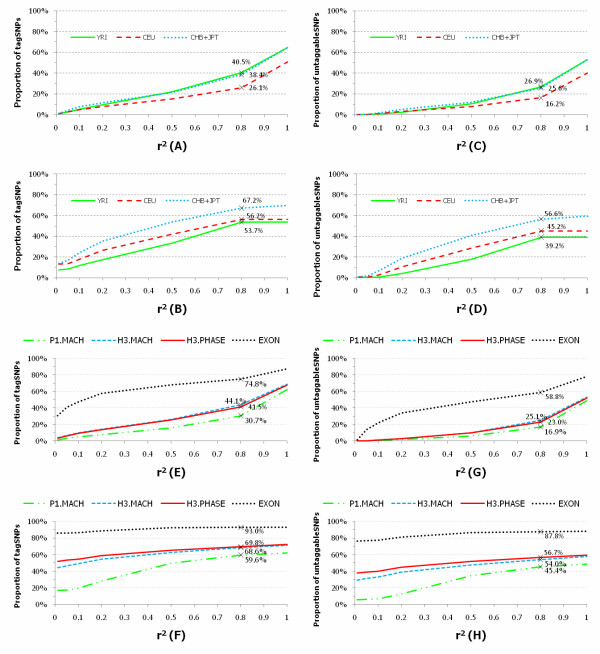
**Proportion of tagged SNPs and untaggable SNPs for the low coverage pilot and imputed dataset**. We assumed the inter-marker distance to be 200 kb and calculated *r*^2 ^between the tag SNP and all nearby SNPs within the distance, and then we plotted the proportions as a function of the *r*^2 ^cut off values for the low coverage pilot dataset in A, B, C and D, and for the imputed CEU dataset in the regions of 382 genes covered by exon pilot project in E,F,G and H, respectively. A) Proportion of tagged common SNPs with 5% < MAF. B) Proportion of tagged low frequency SNPs with 0.5% < MAF ≤ 5%. C) Proportion of untaggable common SNPs with 5% < MAF. D) Proportion of untaggable low frequency SNPs with 0.5% < MAF ≤ 5%. E) Proportion of tagged common SNPs with 5% < MAF. F) Proportion of tagged low frequency SNPs with 0.5% < MAF ≤ 5%. G) Proportion of untaggable common SNPs with 5% < MAF. H) Proportion of untaggable low frequency SNPs with 0.5% < MAF ≤ 5%.

Association studies are still limited by the cost of genotyping the tremendous number of SNPs. To identify a set of informative SNPs which are called tag SNPs by exploiting redundancies among nearby SNPs due to LD may be a choice for genome-wide association studies in practice. It is well known that the tagging approach may substantially improve the genotyping efficiency of common SNPs through the selection of tag SNPs. However, it is unknown if the tagging approach can still dramatically improve the genotyping efficiency of low frequency SNPs. To evaluate the genotyping efficiency of the tagging approach to both common and low frequency SNPs, we used pair-wise methods in which every allele was captured by a single tag at the prescribed *r*^2 ^threshold [10] to select tag SNPs from the low coverage pilot dataset. The results were summarized in Figures 3A-3D which showed the proportion of the tag SNPs with 5% < MAF, the tag SNPs with 0.5% < MAF ≤ 5% and the untaggable SNPs as a function of *r*^2 ^cut off values for the low coverage pilot dataset, respectively. We observed that between 26.1% and 40.5% of the common SNPs used for tag SNPs can capture all common SNPs and between 53.7% and 67.2% of the low frequency SNPs used for tag SNP can capture all low frequency SNPs at *r*^2 ^≥ 0.8 in the CEU, CHB+JPT and YRI samples. Due to greater genetic diversity and weaker LD among low frequency SNPs, more tag SNPs will be required for capturing rare variation in the population. We mark SNPs as untaggable SNPs, if no other SNP within 200 kb has a *r*^2 ^value that is greater than some prespecified threshold. Despite the high density of SNPs in the low coverage pilot dataset, we observed a large proportion of SNPs ranging from 16.2% to 26.9% for common SNPs, and from 39.2% to 56.6% for low frequency SNPs, for which no tag can be identified. The proportion of untaggble low frequency SNPs is approximately twice as high as that of untaggble common SNPs. The proportion of untaggable SNP in the YRI sample is lower than that in the CEU and CHB+JPT samples which may be due to the fact that there are more SNPs with low MAF in the YRI samples than that in the CEU and CHB+JPT samples were removed in the phasing process (Additional file 4, Figure S3).

Next we study the selection of tag SNPs in the regions covered by the exon pilot dataset. To save space we only consider CEU samples. After SNP filtering and removing genes that contained less than 4 called SNPs, a total of 382 genes that contain 2,254 SNPs were remained for data analysis (Additional file 5, Table S2). Since all introns in the 382 genes have not been sequenced, three reference panels: low coverage pilot data in 55 CEU samples (P1.MACH), HapMap 3 unphased (H3.MACH) and phased (H3.PHASE) data [11] in 55 CEU samples were used to impute untyped variants of 85,458 introns as well as 2,254 exons in the regions of 382 genes covered by the exon pilot in 55 CEU samples. All 55 CEU samples were shared by both the low coverage dataset and HapMap 3 dataset. Exon pilot data in the 55 CEU samples without imputation for tag SNP analysis was denoted by EXON. Program MACH was used to impute untyped variants. The results of tag SNP analysis for the exon pilot dataset in the 1000 Genomes Project with imputation from the low coverage pilot dataset in the 1000 Genomes Project and HapMap 3 dataset were summarized in Figure 3E-3H, respectively. These figures showed the proportion of the tag SNPs and untaggable SNPs as a function of *r*^2 ^cut off values. We observe that under imputation by low coverage pilot dataset, 30.7% of the common SNPs used for tag SNPs can capture all common SNPs and 59.6% of the low frequency SNPs used for tag SNP can capture all low frequency SNPs at *r*^2 ^≥ 0.8 in the CEU samples. If the phased and unphased HapMap 3 datasets were used as reference panels then between 41.5% and 44.1% of the common SNPs used for tag SNPs can capture all common SNPs and between 68.6% and 69.8% of the low frequency SNPs used for tag SNP can capture all low frequency SNPs at *r*^2 ^≥ 0.8 in the CEU samples. We also observe that without imputation as high as 74.8% of the common SNPs and 93.0% of the low frequency SNPs used for tag SNPs can capture all common SNPs and low frequency SNPs at *r*^2 ^≥ 0.8 in the CEU samples, respectively. For tag SNP analysis of the exon pilot dataset, we mark SNPs as untaggable SNPs, if no other SNP within the same gene has a *r*^2 ^value that is greater than some prespecified threshold. We found that under imputation with a low coverage pilot as the reference panel, 16.9% of the common SNPs and 45.4% of the low frequency SNPs were untaggable. If the phased and unphased HapMap 3 datasets were used for the reference panel, between 23.0% and 25.1% of the common SNPs and between 54.0% and 56.7% of the low frequency SNPs were untaggable. Without imputation, we observe as high as 58.8% of the common SNPs and 87.8% of the low frequency SNPs, for which no tag SNPs within the same gene can be identified. We also observe that even if the data were imputed the large proportion of low frequency SNPs was untaggable, which will have deep implications for the efficiency of genotyping low frequency tag SNPs.

### Coverage Evaluation

**Figure 4:**
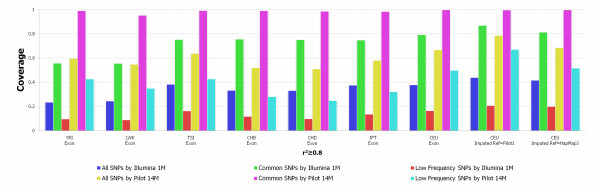
**Coverage of variation**. Coverage of seven populations from the exon pilot between Illumina 1 M and Pilot 14 M when *r*^2 ^≥ 0.8. We imputed two additional CEU populations under the low coverage pilot and HapMap III reference.

To provide information for designing association studies, we evaluate coverage of the genome in the exon pilot dataset by different marker selection strategies including the set of SNPs discovered in the low coverage pilot project (about 14 millions of SNPs) which is referred to as the Pilot 14 M panel, and a commercially available chip, Illumina 1 M. Coverage was evaluated by pair-wise correlation (*r*^2^) between a member of the tag set and its captured SNP [10,12,13]. Figure 4 showed coverage of genomic regions in the exon pilot dataset by six sets of SNP panels. We observed a considerable difference between the coverage for the common and low frequency variation. As expected, 99.0%, 95.1%, 99.7%, 99.1%, 98.9%, 98.5%, and 98.3% of the common variation, in YRI, LWK, CEU, TSI, CHB, CHD and JPT, respectively, were covered by the Pilot 14 M panel even if the coverage was measure by *r*^2 ^≥ 0.8. The SNPs with high frequency which were in the strong LD with the SNPs in the Pilot 14 M panel could be captured indirectly. Although coverage of the common variation by the Pilot 14 M panel is very high, its coverage of low frequency variants was poor. We observed that at *r*^2 ^≥ 0.8, coverage of the low frequency variation in YRI, LWK, CEU, TSI, CHB, CHD and JPT were 43.1%, 35.7%, 49.8%, 43.0%, 28.2%, 24.9% and 32.0%, respectively. This demonstrated that as the number of newly sampled individuals increases, a large proportion of the low frequency variants in the new dataset cannot be captured by any fixed set of selected tag SNPs.

In general, Illumuna 1 M could still capture high proportion of the common variation. The coverage of common variation in YRI, LWK, CEU, TSI, CHB, CHD and JPT by Illumina 1 M was 55.6%, 55.5%, 79.1%, 75.1%, 75.5%, 75.0%, and 74.6%, respectively. However, in contrast to the coverage of the common variation, the majority of the low frequency variation could not be captured by Illumina 1 M. Less than 10% of the low frequency variation in YRI, LWK and CHD samples were covered by Illumina 1 M.

To evaluate the impact of imputation on coverage, we plotted Additional file 6, Figure S4 showing the impact of imputation on the coverage of variation by the Pilot 14 M panel and Illumina 1 M panel, respectively, within the regions of 382 genes (exon pilot) for the CEU samples. We used low frequency pilot data as a reference panel to impute SNPs in the regions of 382 genes (exon pilot) for the CEU samples. We observed few changes in coverage of the common variation by the Pilot 14 M panel in the CEU samples after imputation. However, the coverage of common variation by the Illumina 1 M panel increased from 80% to 87% after imputation. The impact of imputation on the coverage of low frequency variation is significant. We observed that the coverage of low frequency variation by the Pilot 14 M and Illumina 1 M panels was, respectively, increased from 50% and 17% to 67% to 21% after imputation.

### Power Evaluation

The previous power evaluation of association studies has mainly focused on common variation. With the completion of the whole genome sequencing, now it is time to evaluate the power for testing the association of both common and low frequency alleles with the disease under different study designs and disease scenarios. The power of association studies depends on the allele frequencies, penetrance, underlying disease model and the patterns of LD among SNPs. Since the pattern of LD is well modeled by population-genetic simulations [14], we directly use the exon pilot data in the 1000 Genomes Project to carry out power evaluation by simulation (For details, see Methods).

**Figure 5:**
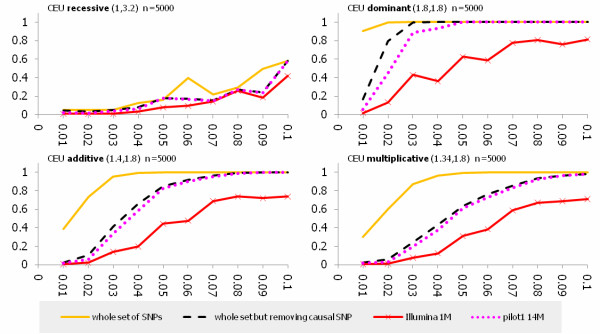
**Power estimations for testing association of low frequency alleles from the CEU population with simulated sample size n = 5000**. The X axis represents allele frequency and the Y axis represents power. Solid line represents the power curve for the whole set of SNPs from the CEU population in the exon pilot, the dashed line represent the power curve for the set of all SNPs from the CEU population in the exon pilot except for the putative causal SNP. The dotted line represent the power curve for the low coverage Pilot 14 M dataset, and the solid line with the star represents the power curve for the Illumina 1 M. Values in parentheses are the heterozygous and homozygous relative risk, respectively. Only putative causal SNPs in the low frequency region are presented.

Power was calculated at the significance level α = 0.05. We evaluate four whole-genome products: the set of all SNPs in the exon pilot dataset, the set of all SNPs except for the putative causal SNP, low coverage Pilot 14 M, and Illumina 1 M. The power was averaged for each allele frequency across 382 genes in the exon pilot dataset. The estimates of power for testing association of the low frequency allele under the dominant, additive, multiplicative and recessive disease models in the CEU was shown in Figure 5 (n = 5000) and Additional file 7, Figure S5 (n = 3000), respectively, where the first number in parenthesis was the heterozygous relative risk and the second number was the homozygous relative risk. We observed several remarkable features. First, using data from all SNPs in the exon pilot dataset achieved the greatest power, and followed by the set of all SNPs except for the putative causal SNP, Pilot 14 M and Illumina 1 M. The power curves using the set of all SNPs except for the putative causal SNP and using Pilot 14 M were similar. Second, power under the dominant disease models was the largest and power under the receive models was the smallest. Third, the risk allele frequency and genotype relative risks markedly affected the power. We observed that when the MAF was 0.01, even if the sample size was increased to 5,000, both heterozygous and homozygous relative risks were increased to 1.8, the power using data from all SNPs still could not be higher than 0.5 except for dominant disease models. Fourth, differences in power between using the data from all SNPs and low coverage Pilot 14 M was remarkable. When minor allele frequency was 0.01, the power using the Pilot 14 M for association studies was extremely low. Indeed, the power of the Pilot 14 M for the dominant (both heterozygous and homozygous relative risks were 1.8), additive (heterozygous relative risk 1.4 and homozygous relative risk 1.8), multiplicative (heterozygous relative risk 1.34 and homozygous relative risk 1.8) and recessive models (heterozygous relative risk 1 and homozygous relative risk 3.2) with sample size 5000 was 0.054, 0.016, 0.015 and 0.022, respectively. The power of Pilot 14 M under the above four disease models for the MAF of 0.02 was 0.45, 0.058, 0.031 and 0.018, respectively. The only difference between these two datasets was that the samples in the exon pilot dataset were 90 individuals and samples in the low coverage Pilot 14 M was 60 individuals. Here we should emphasize that the allele frequency spectrum for the 60 sampled individuals will be largely different from the allele frequency spectrum for the 90 sampled individuals (60 previous sampled individuals plus additional 30 sampled individuals). In this case, we can expect that the low coverage Pilot 14 M will definitely have no power to detect association of low frequency alleles. Fifth, the power of commercial arrays for testing association of low frequency alleles in all cases was extremely low.

The patterns of power for the samples from the CHD for testing association of low frequency variation were similar to that for the samples from the CEU (Fig. S6). The power for testing association of both common and low frequency alleles in the CEU and CHD was plotted in Additional file 8, Figure S6 and Additional file 9, Figure S7. As expected, when the MAF increases, the power will increase and using whole dataset or potential low coverage Pilot 14 M array for testing the association of common alleles can reach a high power for some disease models. When sample sizes were 5,000, the power of using the whole exon pilots dataset and potential Pilot 14 M array in the CEU samples under the dominant, additive, multiplicative and recessive models with MAF = 5% was 1, 0.997, 0.990, 0.162, and 1, 0.827, 0.612, 0.175, respectively. The power of Pilot 14 M under the above four disease models in the CHD was 1, 0.998, 0.987, 0.174, and 0.816, 0.643, 0.460 and 0.0815, respectively. Except for the recessive models, the power of using the potential low coverage Pilot 14 M array for testing association of alleles with MAF ≥ 7% in both CEU and CHD samples were higher than 0.80. This demonstrates that the potential low coverage Pilot 14 M array can be used for whole genome association studies of common variation.

**Figure 6:**
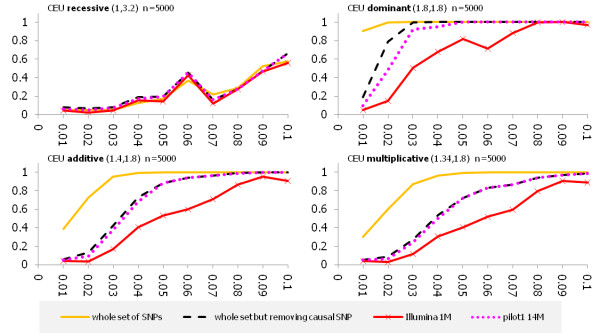
**Power estimations for testing association of low frequency allele from the CEU population with simulated sample size n = 5000 after imputation**. The X axis represents allele frequency and the Y axis represents power. The solid line represents the power curve for the whole set of SNPs from 382 genes of the CEU population in the exon pilot, the dashed line represent the power curve for the set of all SNPs from 382 genes of CEU population in the exon pilot except for the putative causal SNP. The dotted lines represent the power curve for the low coverage Pilot 14 M dataset, and the solid line with the star represents the power curve for the Illumina 1 M. Values in parentheses are the heterozygous and homozygous relative risk, respectively. Only putative causal SNPs in the low frequency region are presented.

Imputation can increase the power of the association test. To save space, we only studied the impact of imputation on the power for the CEU samples. However, the pattern of improvement of the power by imputation for other populations was similar. Imputation was performed as before. The power for testing the association of low frequency and common SNPs in the CEU samples after imputation was shown in Figures 6 (n = 5000), Additional file 10, Figure S8 (n = 3000) and Additional file 11, Figure S9 (n = 1000), respectively. From these Figures we see that if the whole exon pilot dataset was used we observed no improvements of imputation on the power. However, if the low coverage Pilot 14 M array and Illuimina 1 M array were used for association studies, these Figures showed that imputation can increase the power of the statistics for testing association of both common and low frequency variations. The effect of imputation on the power depended on the relative risks, disease susceptibility allele frequencies and disease models. We observed that imputation had less impact on the power of the test under the recessive models than that under the other disease models.

## Discussion

For the past several years, there have been debates on what variants, common variants or rare variants cause complex diseases. However, it is gaining common consensus that wide the allelic spectrum of genetic variants would underlie the development of diseases [15]. Emerging 'next-generation' sequencing (NGS) technologies enable sequencing individual genomes and have the potential to discover the entire spectrum of sequence variations in a sample of well-phenotyped individuals. Advances in sequencing technologies are revolutionizing genetic studies of complex diseases and provide unprecedented new opportunities to test for association of the entire spectrum of genetic variants with the disease. The challenge now is what paradigm for the next generation GWAS which are based on NGS should be developed. At least two distinct study designs: (1) chips for genotyping variants and (2) sequencing genome for investigating variants have been proposed for next generation GWAS. The current chip-based GWAS paradigm which catalogue common variants with 5% < MAF and genotype them using chips is mainly designed for testing the association of common SNPs with disease. Remarkable features of common genetic variants are that LD between common variants is strong and the full extent of common variants can be discovered with a limited number of samples. The chip approach is to extend the current GWAS for common variants to the low frequency variants (0.5% < MAF ≤ 5%) and rare variants (MAF ≤ 0.5%). However, the LD between rare variants and between rare and common variants is often weak. The number of novel rare variants increases when the number of sampled individuals increases. Chip-based GWAS for testing the association of rare variants with the disease has serious limitations. Recently, the paradigm of association studies is being shifted to sequence-based association studies. Sequencing approach is to sequence the whole exome or entire genome to capture low frequency and rare variants instead of genotyping a catalogue of variants [4]. The 1000 Genomes Project uses the next-generation sequencing technologies to generate genome-wide resources with a comprehensive survey of the entire allelic spectrum of genetic variation. In this report we used this rich resource to evaluate different study designs for the next generation GWAS and addressed several issues in the whole-genome and whole-exome sequencing-based association studies.

First, we observed that when the number of typed individuals increases, the proportion of low frequency SNPs dramatically increases. This implies that the 1000 Genome Project is not able to identify all low frequency and rare genetic variants and catalogue them. Sampling different sets of individuals may have different set of low frequency and rare variants. The newly presented low frequency and rare variants in the samples may not be covered by any commercial arrays with the set of fixed SNPs. This implies that through GWAS and imputation of the 1000 Genome Project variants we may still miss causal variants in association studies. Second, we investigated the pattern of LD. We found that the LD level between the low frequency and low frequency alleles, particularly low frequency and common alleles is low, which implies that using variants in the chips has diminished power to detect causal SNPs with low allele frequency which are not printed in the Chips. The efficiency of tag SNPs that represent all variants in the genomic regions of interest depends on the level of LD between SNPs. With weak LD among SNPs we need to select large proportions of SNPs as tag SNPs to capture most of the genomic variation. In this case, the effect of using tag SNPs to reduce genotyping cost is poor. Therefore, the tag SNP approach which has been successfully used in the current GWAS for testing association of common variants may be highly inefficient for testing association of low frequency and rare variants with the disease. Third, we found that although coverage of common variation in the exon pilot dataset by the variants in the low coverage pilot data is very high, its coverage of low frequency variants by the variants in the low coverage pilot dataset was very poor. This showed that a large proportion of the novel variants with low frequency generated by sequencing new individual cannot be covered by the next generation genotyping arrays with a set of fixed variants discovered by the 1000 Genomes Project. Fourth, next generation genotyping arrays such as the potential Pilot 14 M array which capture a considerable portion of genomic common variation have high power to detect association of the common variants with the disease, but its power to identify low frequency or rare variants which are associated with the disease is low. Fifth, to test association of low frequency or rare variants still raises great challenges. We assessed the power of various strategies for genome-wide exon association studies. Although using all the data generated by the exon pilot project has reasonable power to detect association of low frequency variants under the dominant, additive and multiplicative models with typical genotype relative risks, the power of the potential Pilot 14 M array to detect association of low frequency variants is low. When sample sizes increase, more and more novel rare variants will not be included and captured by the SNPs in the Pilot 14 M array. It is anticipated that many causative low frequency and rare variants will be missing in GWAS by the Pilot 14 M. This raises the concern of feasibility of using next generation genotyping arrays for association studies of low frequency and rare variants. Imputation can improve coverage, reduce the number of tag SNPs and increase the power. However, the power increased by imputation is limited. Imputation is highly unlikely to change the above statements. Our results clearly demonstrate that only sequencing the whole genome can identify all the causative variants including both common and rare variants. NGS technologies represent a paradigm shift in association studies of common diseases.

The 1000 Genomes Project dramatically expands the genome-wide sources of all types of genetic variation. The data generated by the 1000 Genomes Project provide rich information for the evaluation of various strategies and designs for association studies of the entire allelic spectrum of genetic variation. The results presented in this report are preliminary. Systematic, definite and more powerful evaluation of association study strategies and designs awaits more expanded datasets including the complete 1000 Genomes Project dataset and the results of further studies. However, next generation sequencing technologies open a new exciting avenue to decipher the path from genomic information to phenotypes.

## Conclusions

NGS technologies with faster, cheaper and more accurate sequencing represent a paradigm shift in measuring genomic variants. It will generate unprecedented massive data and have the potential to discover the entire spectrum of genetic variation. NGS offers a rich resource for dissecting genetic structure of common diseases, but also presents formidable challenges to data analysis. Systematic, definite and more powerful evaluation of association study strategies and designs awaits more expanded datasets including the complete 1000 Genomes Project dataset and the results of further studies.

## Methods

### Characterization of LD pattern

LD was measured by the squared correlation coefficient *r*^2 ^between pairs of SNPs. For each SNP within a chromosome which was taken as a target SNP in turn, we calculated the squared correlation coefficient *r*^2 ^between the target SNP and all its nearby SNPs within an inter-marker distance of 50 kb, 100 kb and 200 kb. The total number of pairs of SNPs across autosomal chromosomes was the summation of the number of all possible pairs of SNPs within each chromosome. The LD pattern was described as the proportion of pairs of SNPs with the *r*^2 ^greater or less than the specified threshold. We used the PHASE program [16] with 30 iterations and 30 burn-in to estimate the individual haplotype.

### Selection of tag SNPs

First select a single SNP exceeding the *r*^2 ^threshold with the maximum number of other SNPs within a specified inter-marker distance [17]. The selected SNP which is referred to as a tag SNP and all other associated SNPs (tagged SNPs) are grouped as a bin of associated SNPs. Repeat the process for the remaining SNPs until all SNPs are tagged.

### Imputation

The MACH program [18] with the "mle" and "greedy" options selected was used to impute untyped variants of 85,458 introns as well as 2,254 exons in the regions of 382 genes (Additional file 1, Table S1) covered by the exon pilot data in 55 CEU samples. Iterations were performed 100 times. The squared correlation between the true genotype and the imputed genotype, averaged across all imputed SNPs was selected as the criterion of imputation merit. The low coverage pilot data in 55 CEU samples (P1.MACH) which were shared by both the low coverage datasets and HapMap 3 dataset, HapMap 3 unphased (H3.MACH) and phased (H3.PHASE) data in 55 CEU samples were used as reference panels.

### Coverage

The set of SNPs discovered in the low coverage pilot project (about 14 millions of SNPs) which is referred to as Pilot 14 M, and the set of SNPs in the commercially available Illumina 1 M chip were used as a set of tag SNPs. Coverage of the exon regions in the exon pilot dataset (total 1.43 Mb) was evaluated by the pair-wise correlation between a member of the tag set and its captured SNP and defined as the proportion of SNPs within the exon regions in the exon pilot dataset, captured by the set of tag SNPs at the specified threshold on the value of *r*^2^. We estimated coverage of the exon regions for seven populations by unimputed data and coverage for the CEU samples by imputed data.

### Power calculation

We used the simulation methods [19] for power calculation. Each SNP site in the exon regions in the exon pilot dataset was taken as a causal SNP in turn. For every causal SNP we simulated two case-control panels: (1) 3,000 cases and 3,000 controls and (2) 5,000 cases and 5,000 controls by resampling chromosomes with exon pilot data from unrelated individuals in the CEU (n = 180) or in the CHD (n = 214). The Chi-square statistic with two degrees of freedom was used to test association of a single putative causal variant. We simulated cases under four disease models: dominant, additive, multiplicative and recessive models with prevalence of 0.01. To select appropriate relative risk for power simulation we studied 1,256 common disease susceptibility loci from website (http://www.genome.gov/gwastudies). The average value and median relative risk for common diseases were 1.5 and 1.183, and their square was 2.25 and 1.4, respectively. Therefore, we selected 1.4 and 1.8 as the relative risks for the homozygous genotype for the common variants under all four disease models. To increase the power, we selected 1.8 as the relative risks for the homozygous genotype for the low frequency variants under the dominant, additive and multiplicative disease models, and 3.2 for the recessive disease model. The power was calculated at the significance level of 0.05 and plotted as a function of the frequency of disease susceptibility allele.

## Authors' contributions

HS and YZ wrote computer scripts and conducted analysis. LJ participated in the study design. MX designed study and wrote the manuscript. All the authors discussed the results, read, and approved the final manuscript.

## Supplementary Material

Additional file 1 **Figure S1 - Proportion of low frequency and common variants**. Click here for file

Additional file 2 **Table S1 - The number of variants in the low coverage and exon pilot datasets in 1000 Genomes Project**. Click here for file

Additional file 3 **Figure S2 - Pattern of LD between common and common variants based on the HapMap II (r22) dataset**. The proportions of pair-wise SNPs with *r*^2 ^between common and common SNPs in five intervals of *r*^2 ^for the HapMap II (r22) dataset, and then we plotted graphs under three inter-marker distances, A) 50 kb, B) 100 kb and C) 200 kb where *r*^2 ^between the target SNP and its all nearby SNPs within the distance was calculated. Click here for file

Additional file 4 **Figure S3 - Allele frequency distribution for low coverage pilot autosomal SNPs after PHASE**. The MAF distribution from 0 to 0.5 is shown. The red dashed line shows the MAF distribution expected for the standard neutral population model with a constant population size and random mating, the blue solid line shows the MAF distribution for all the observed variants, and the green dotted line shows the MAF distribution for the observed non-synonymous alleles. Click here for file

Additional file 5 **Table S2 - A total of 382 genes in the exon pilot dataset**. Click here for file

Additional file 6 **Figure S4 - Impact of imputation on the coverage**. A) Impact of imputation on the coverage of all variation by the Pilot 14 M panel within the regions of 382 genes (exon pilot) for the CEU samples. B) Impact of imputation on the coverage of common variation by the Pilot 14 M panel within the regions of 382 genes (exon pilot) for the CEU samples. C) Impact of imputation on the coverage of low frequency variation by Pilot the 14 M panel within the regions of 382 genes (exon pilot) for the CEU samples. D) Impact of imputation on the coverage of all variation by the Illumina 1 M panel within the regions of 382 genes (exon pilot) for the CEU samples. E) Impact of imputation on the coverage of common variation by the Illumina 1 M panel within the regions of 382 genes (exon pilot) for the CEU samples. F) Impact of imputation on the coverage of low frequency variation by the Illumina 1 M panel within the regions of 382 genes (exon pilot) for the CEU samples. Click here for file

Additional file 7 **Figure S5 - Power estimations for testing association of the low frequency allele from the CEU population with simulated sample size n = 3000**. The X axis represents allele frequency and the Y axis represents power. The solid line represents the power curve for the whole set of SNPs from the CEU population in the exon pilot, the dashed line represent the power curve for the set of all SNPs from the CEU population in the exon pilot except for the putative causal SNP, the dotted line represents the power curve for the low coverage Pilot 14 M dataset, and the solid line with the star represents the power curve for the Illumina 1 M. Values in parentheses are the heterozygous and homozygous relative risk, respectively. All putative causal SNPs in the low frequency region are presented. Click here for file

Additional file 8 **Figure S6 - Power estimations for testing association of the whole frequency allele from the CEU population**. The X axis represents allele frequency and the Y axis represents power. The solid line represents the power curve for the whole set of SNPs from the CEU population in the exon pilot, the dashed line represents the power curve for the set of all SNPs from the CEU population in the exon pilot except for the putative causal SNP, the dotted line represents the power curve for the low coverage Pilot 14 M dataset, and the solid line with the star represents the power curve for the Illumina 1 M. Values in parentheses are the heterozygous and homozygous relative risk, respectively. All putative causal SNPs in the whole frequency region are presented. A) Estimation under the recessive model. B) Estimation under the dominant model. C) Estimation under the additive model. D) Estimation under the multiplicative model. Click here for file

Additional file 9 **Figure S7 - Power estimations for testing association of the whole frequency allele from the CHD population**. The X axis represents allele frequency and the Y axis represents power. The solid line represents the power curve for the whole set of SNPs from the CHD population in the exon pilot, the dashed line represent the power curve for the set of all SNPs from the CHD population in the exon pilot except for the putative causal SNP, the dotted line represents the power curve for the low coverage Pilot 14 M dataset, and the solid line with the star represents the power curve for the Illumina 1 M. Values in parentheses are the heterozygous and homozygous relative risk, respectively. All putative causal SNPs in whole frequency region are presented. A) Estimation under the recessive model. B) Estimation under the dominant model. C) Estimation under the additive model. D) Estimation under the multiplicative model. Click here for file

Additional file 10 **Figure S8 - Power estimations for testing association of the low frequency allele from the CEU population with simulated sample size n = 3000 after imputation**. The X axis represents allele frequency and the Y axis represents power. The solid line represents the power curve for the whole set of SNPs from 382 genes of the CEU population in the exon pilot, the dashed line represents the power curve for the set of all SNPs from 382 genes of the CEU population in the exon pilot except for the putative causal SNP, the dotted line represents the power curve for the low coverage Pilot 14 M dataset, and the solid line with the star represents the power curve for the Illumina 1 M. Values in parentheses are the heterozygous and homozygous relative risk, respectively. All putative causal SNPs in the low frequency region are presented. Click here for file

Additional file 11 **Figure S9 - Power estimations for testing association of whole frequency allele from CEU population after imputation**. The X axis represents allele frequency and the Y axis represents power. The solid line represents the power curve for the whole set of SNPs from 382 genes of the CEU population in the exon pilot, the dashed line represents the power curve for the set of all SNPs from 382 genes of the CEU population in the exon pilot except for the putative causal SNP, the dotted line represents the power curve for the low coverage Pilot 14 M dataset, and the solid line with the star represents the power curve for the Illumina 1 M. Values in parentheses are the heterozygous and homozygous relative risk, respectively. All putative causal SNPs in whole frequency region are presented. A) Estimation under the recessive model. B) Estimation under the dominant model. C) Estimation under the additive model. D) Estimation under the multiplicative model. Click here for file
