# Supplementary material for: Implication of next-generation sequencing on association studies
Source: BMC Genomics. 2011 Jun 17;12:322. doi: 10.1186/1471-2164-12-322 (PMC3148210; doi:10.1186/1471-2164-12-322)
Supplement: Additional file 2 — Table S1 - The number of variants in the low coverage and exon pilot datasets in 1000 Genomes Project. [file 1471-2164-12-322-S2.DOC]

|  | Variants | | | Density (per 1kb) | | |
| --- | --- | --- | --- | --- | --- | --- |
| Population | All# | Rare# | Common# | All | Rare | Common |
| YRI.low coverage | 9,721,002 | 3,131,555 | 6,589,447 | 3.49 | 1.12 | 2.37 |
| CEU.low coverage | 7,147,833 | 2,324,350 | 4,823,483 | 2.57 | 0.83 | 1.73 |
| CHB+JPT.low coverage | 5,042,654 | 1,049,336 | 3,993,318 | 1.81 | 0.38 | 1.43 |
| YRI.exon pilot | 3,593 | 2,500 | 1,093 | 1.55 | 1.08 | 0.47 |
| LWK.exon pilot | 5,632 | 3,783 | 1,849 | 2.43 | 1.63 | 0.80 |
| CEU.exon pilot | 2,674 | 1,765 | 909 | 1.15 | 0.76 | 0.39 |
| TSI.exon pilot | 2,689 | 1,687 | 1,002 | 1.16 | 0.73 | 0.43 |
| CHB.exon pilot | 2,467 | 1,632 | 835 | 1.06 | 0.70 | 0.36 |
| CHD.exon pilot | 3,855 | 2,493 | 1,362 | 1.66 | 1.07 | 0.59 |
| JPT.exon pilot | 2,001 | 1,212 | 789 | 0.86 | 0.52 | 0.34 |

Table S1. Number of variants in the low coverage and exon pilot datasets in 1000 Genomes Project
