# Supplementary material for: Implication of next-generation sequencing on association studies
Source: BMC Genomics. 2011 Jun 17;12:322. doi: 10.1186/1471-2164-12-322 (PMC3148210; doi:10.1186/1471-2164-12-322)
Supplement: Additional file 5 — Table S2 - A total of 382 genes in the exon pilot dataset. [file 1471-2164-12-322-S5.DOC]

**Table S2.** A total of 382 genes in the exon pilot dataset.

| chr | symbol | chr | symbol | chr | symbol | chr | symbol |
| --- | --- | --- | --- | --- | --- | --- | --- |
| 1 | WDR8 | 3 | TRPC1 | 9 | NOL6 | 15 | ATP10A |
| 1 | TNFRSF25 | 3 | LAMP3 | 9 | C9orf131 | 15 | EPB42 |
| 1 | ARHGEF10L | 3 | PSMD2 | 9 | SMC5 | 15 | TGM5 |
| 1 | ACTL8 | 3 | MASP1 | 9 | INVS | 15 | MFAP1 |
| 1 | KIF17 | 3 | LEPREL1 | 9 | OR13C5 | 15 | SLC28A2 |
| 1 | C1orf63 | 3 | BDH1 | 9 | DENND1A | 15 | ATP8B4 |
| 1 | C1orf94 | 4 | C4orf50 | 9 | PKN3 | 15 | ALDH1A2 |
| 1 | CLSPN | 4 | AFAP1 | 9 | FAM166A | 15 | VPS13C |
| 1 | ZNF642 | 4 | ACOX3 | 10 | AKR1CL1 | 15 | PAQR5 |
| 1 | ST3GAL3 | 4 | SLC34A2 | 10 | PPYR1 | 15 | ALPK3 |
| 1 | PIK3R3 | 4 | RBPJ | 10 | FRMPD2 | 15 | SLC28A1 |
| 1 | DMBX1 | 4 | ARHGAP24 | 10 | ERCC6 | 15 | AKAP13 |
| 1 | ELAVL4 | 4 | FAM13A | 10 | CHAT | 15 | TTLL13 |
| 1 | FGGY | 4 | PDLIM5 | 10 | ASAH2 | 15 | LASS3 |
| 1 | NFIA | 4 | CENPE | 10 | H2AFY2 | 16 | BAIAP3 |
| 1 | PDE4B | 4 | C4orf33 | 10 | SGPL1 | 16 | HAGH |
| 1 | MSH4 | 4 | PCDH10 | 10 | GPR120 | 16 | MKL2 |
| 1 | ST6GALNAC3 | 4 | TKTL2 | 10 | CYP2C18 | 16 | ABCC6 |
| 1 | PTGFR | 4 | ANP32C | 10 | SORBS1 | 16 | USP31 |
| 1 | GBP6 | 4 | TLL1 | 10 | DNMBP | 16 | COG7 |
| 1 | PSRC1 | 4 | PALLD | 10 | NT5C2 | 16 | DCTN5 |
| 1 | AMPD2 | 4 | NEIL3 | 10 | ZDHHC6 | 16 | PRKCB |
| 1 | ST7L | 5 | PLEKHG4B | 10 | CASP7 | 16 | GPT2 |
| 1 | MOV10 | 5 | SLC6A3 | 10 | ATRNL1 | 16 | CCDC135 |
| 1 | PPM1J | 5 | MTRR | 10 | PNLIPRP1 | 16 | C16orf48 |
| 1 | TRIM33 | 5 | COL4A3BP | 10 | PDZD8 | 16 | CDH1 |
| 1 | TARS2 | 5 | F2RL1 | 10 | TACC2 | 16 | NQO1 |
| 1 | ARNT | 5 | HISPPD1 | 10 | CLRN3 | 16 | ZC3H18 |
| 1 | CRNN | 5 | ZNF474 | 10 | LRRC27 | 16 | SPG7 |
| 1 | ADAM15 | 5 | PHF15 | 11 | RIC8A | 17 | GLOD4 |
| 1 | CD1C | 5 | SLC23A1 | 11 | ANO9 | 17 | TRPV3 |
| 1 | OR10J1 | 5 | PCDHB11 | 11 | MUPCDH | 17 | ARHGEF15 |
| 1 | SLAMF8 | 5 | PCDHG+ | 11 | OR51B4 | 17 | GLP2R |
| 1 | NCSTN | 5 | KIAA0141 | 11 | OR52H1 | 17 | SREBF1 |
| 1 | USP21 | 5 | ABLIM3 | 11 | OR56A4 | 17 | CCT6B |
| 1 | ADAMTS4 | 5 | SLC26A2 | 11 | OR2D3 | 17 | SLFN13 |
| 1 | LRRC52 | 5 | CCDC69 | 11 | GALNTL4 | 17 | C17orf66 |
| 1 | C1orf9 | 5 | FOXI1 | 11 | KCNJ11 | 17 | LYZL6 |
| 1 | SLC9A11 | 5 | STK10 | 11 | OR8H1 | 17 | AATF |
| 1 | GPR37L1 | 5 | PCDH24 | 11 | AHNAK | 17 | PIP4K2B |
| 1 | OPTC | 5 | ZFP2 | 11 | SLC3A2 | 17 | PLXDC1 |
| 1 | PIK3C2B | 6 | IRF4 | 11 | PYGM | 17 | IKZF3 |
| 1 | LRRN2 | 6 | RREB1 | 11 | CTSW | 17 | ZPBP2 |
| 1 | NUAK2 | 6 | RIOK1 | 11 | GAB2 | 17 | KRT27 |
| 1 | LGTN | 6 | SLC17A3 | 11 | SYTL2 | 17 | KRT9 |
| 1 | LAMB3 | 6 | PGBD1 | 11 | MTMR2 | 17 | SKAP1 |
| 1 | IRF6 | 6 | HCG9 | 11 | TMEM133 | 17 | FAM117A |
| 1 | TMEM63A | 6 | BAT2 | 11 | GRIA4 | 17 | STXBP4 |
| 1 | URB2 | 6 | PSMB8 | 11 | EXPH5 | 17 | TEX14 |
| 1 | ARID4B | 6 | SLC26A8 | 11 | PPP2R1B | 17 | HEATR6 |
| 2 | ODC1 | 6 | KLHL31 | 11 | TMEM225 | 17 | EFCAB3 |
| 2 | SLC4A1AP | 6 | MRAP2 | 12 | CLEC2D | 17 | CD300LB |
| 2 | QPCT | 6 | MDN1 | 12 | PRR4 | 18 | EMILIN2 |
| 2 | ATL2 | 6 | FBXL4 | 12 | TMEM106C | 18 | KIAA0802 |
| 2 | FSHR | 6 | SLC16A10 | 12 | ADCY6 | 18 | ANKRD12 |
| 2 | BCL11A | 6 | MCM9 | 12 | KRT73 | 18 | TXNDC2 |
| 2 | LONRF2 | 6 | AKAP7 | 12 | AMHR2 | 18 | APCDD1 |
| 2 | TMEM177 | 6 | VNN3 | 12 | OR6C76 | 18 | ZNF521 |
| 2 | MBD5 | 6 | MYB | 12 | SRGAP1 | 18 | DSC3 |
| 2 | GPD2 | 6 | FUCA2 | 12 | KRR1 | 18 | DSC1 |
| 2 | ACVR1C | 6 | UTRN | 12 | ALX1 | 18 | ZBTB7C |
| 2 | LY75 | 6 | TIAM2 | 12 | ATP2B1 | 18 | MBD1 |
| 2 | NEUROD1 | 6 | AGPAT4 | 12 | EPYC | 18 | CDH7 |
| 2 | WDR75 | 7 | PMS2 | 12 | PLEKHG7 | 19 | PTPRS |
| 2 | MYO1B | 7 | NPSR1 | 12 | PLXNC1 | 19 | LASS4 |
| 2 | SDPR | 7 | NPC1L1 | 12 | C12orf63 | 19 | SLC44A2 |
| 2 | CASP10 | 7 | PON2 | 12 | SART3 | 19 | NOTCH3 |
| 2 | XRCC5 | 7 | ACHE | 12 | TRPV4 | 19 | OR10H3 |
| 2 | RNF25 | 7 | FBXL13 | 12 | CCDC63 | 19 | NPHS1 |
| 2 | TTLL4 | 7 | RELN | 12 | TRAFD1 | 19 | ZNF540 |
| 2 | SP110 | 7 | WNT16 | 12 | ANAPC5 | 19 | TIMM50 |
| 2 | GIGYF2 | 7 | PAX4 | 12 | SBNO1 | 19 | HIF3A |
| 2 | UGT1A+ | 7 | FAM71F1 | 12 | DHX37 | 19 | PNMAL1 |
| 2 | SPP2 | 7 | SMO | 12 | POLE | 19 | ZNF616 |
| 2 | COL6A3 | 7 | CPA1 | 12 | CHFR | 19 | PRKCG |
| 2 | MYEOV2 | 7 | CREB3L2 | 13 | ZMYM5 | 19 | TMC4 |
| 2 | MTERFD2 | 7 | ZNF282 | 13 | TNFRSF19 | 19 | NLRP11 |
| 3 | CRELD1 | 7 | GIMAP7 | 13 | FLT1 | 19 | ZNF264 |
| 3 | COLQ | 7 | ACCN3 | 13 | B3GALTL | 19 | ZNF329 |
| 3 | THRB | 7 | NOM1 | 13 | TRPC4 | 20 | UBOX5 |
| 3 | CRTAP | 8 | LPL | 13 | FREM2 | 20 | C20orf26 |
| 3 | PLCD1 | 8 | CDCA2 | 13 | PIBF1 | 20 | CEP250 |
| 3 | SLC22A14 | 8 | PBK | 14 | FLJ10357 | 20 | WFDC3 |
| 3 | SETD2 | 8 | NRG1 | 14 | OXA1L | 20 | ZSWIM1 |
| 3 | KIF9 | 8 | RNF122 | 14 | ACIN1 | 20 | SULF2 |
| 3 | NCKIPSD | 8 | SLC20A2 | 14 | EFS | 20 | CYP24A1 |
| 3 | ARHGEF3 | 8 | CYP7B1 | 14 | RIPK3 | 20 | RTEL1 |
| 3 | GPR128 | 8 | XKR9 | 14 | BAZ1A | 21 | SYNJ1 |
| 3 | NFKBIZ | 8 | TMEM67 | 14 | SOCS4 | 21 | IFNGR2 |
| 3 | CBLB | 8 | RAD54B | 14 | MUDENG | 21 | ETS2 |
| 3 | IFT57 | 8 | COL14A1 | 14 | KCNH5 | 21 | AGPAT3 |
| 3 | MORC1 | 8 | GSDMC | 14 | C14orf145 | 22 | SEC14L3 |
| 3 | GOLGB1 | 8 | TG | 14 | C14orf159 | 22 | LIMK2 |
| 3 | PODXL2 | 9 | KANK1 | 14 | SERPINA11 | 22 | LARGE |
| 3 | TMCC1 | 9 | VLDLR | 14 | AK7 |  |  |
| 3 | TRIM42 | 9 | RCL1 | 14 | HSP90AA1 |  |  |
