# Supplementary figures and images for: Implication of next-generation sequencing on association studies
Source: BMC Genomics. 2011 Jun 17;12:322. doi: 10.1186/1471-2164-12-322 (PMC3148210; doi:10.1186/1471-2164-12-322)

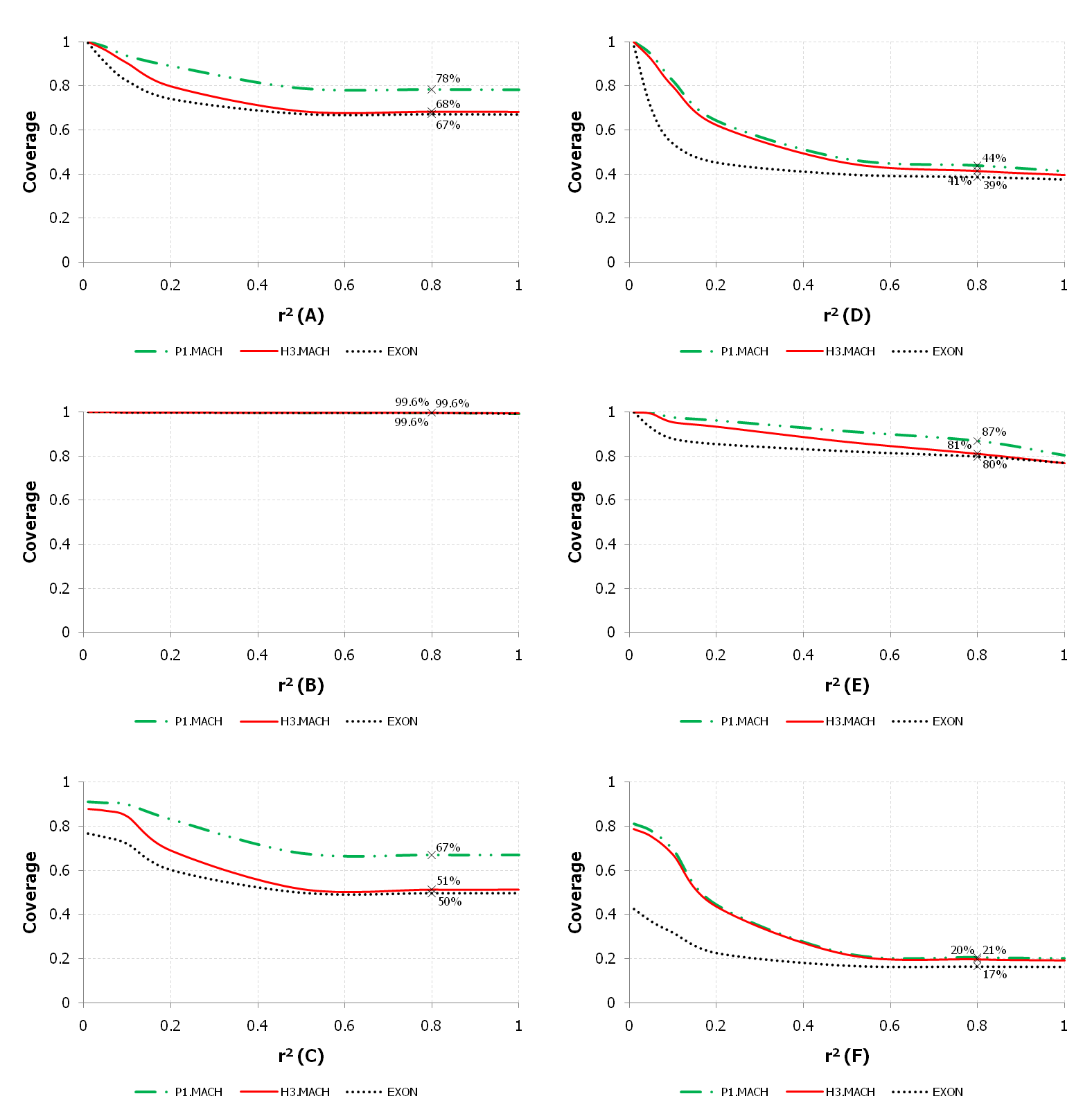

Supplement: Additional file 6 — Figure S4 - Impact of imputation on the coverage. A) Impact of imputation on the coverage of all variation by the Pilot 14 M panel within the regions of 382 genes (exon pilot) for the CEU samples. B) Impact of imputation on the coverage of common variation by the Pilot 14 M panel within the regions of 382 genes (exon pilot) for the CEU samples. C) Impact of imputation on the coverage of low frequency variation by Pilot the 14 M panel within the regions of 382 genes (exon pilot) for the CEU samples. D) Impact of imputation on the coverage of all variation by the Illumina 1 M panel within the regions of 382 genes (exon pilot) for the CEU samples. E) Impact of imputation on the coverage of common variation by the Illumina 1 M panel within the regions of 382 genes (exon pilot) for the CEU samples. F) Impact of imputation on the coverage of low frequency variation by the Illumina 1 M panel within the regions of 382 genes (exon pilot) for the CEU samples. [file 1471-2164-12-322-S6.TIFF]

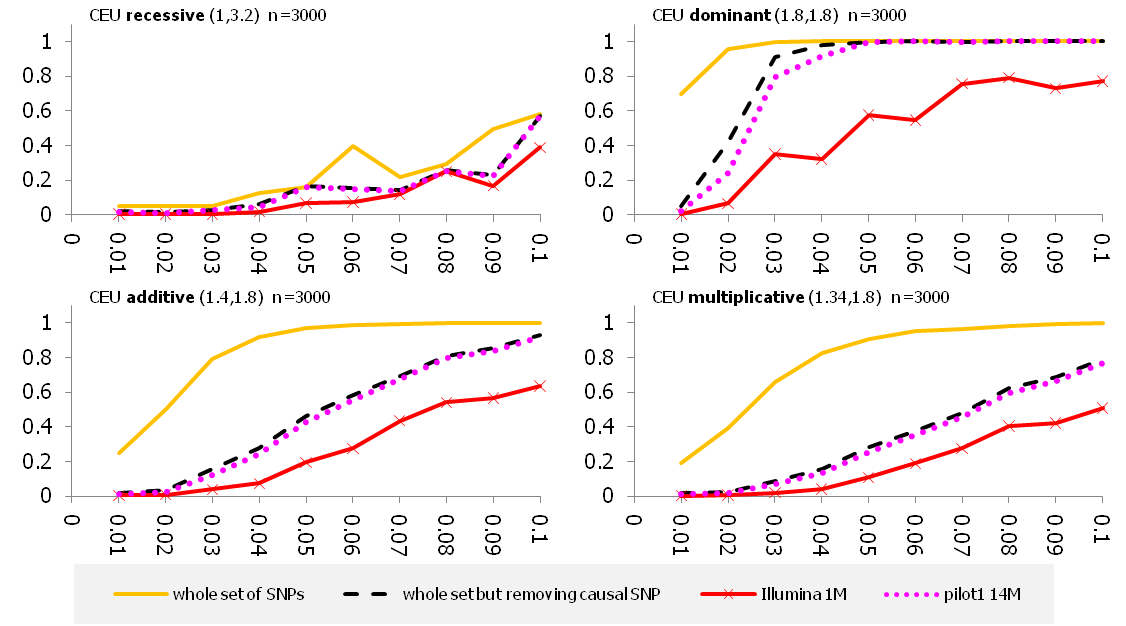

Supplement: Additional file 7 — Figure S5 - Power estimations for testing association of the low frequency allele from the CEU population with simulated sample size n = 3000. The X axis represents allele frequency and the Y axis represents power. The solid line represents the power curve for the whole set of SNPs from the CEU population in the exon pilot, the dashed line represent the power curve for the set of all SNPs from the CEU population in the exon pilot except for the putative causal SNP, the dotted line represents the power curve for the low coverage Pilot 14 M dataset, and the solid line with the star represents the power curve for the Illumina 1 M. Values in parentheses are the heterozygous and homozygous relative risk, respectively. All putative causal SNPs in the low frequency region are presented. [file 1471-2164-12-322-S7.TIFF]

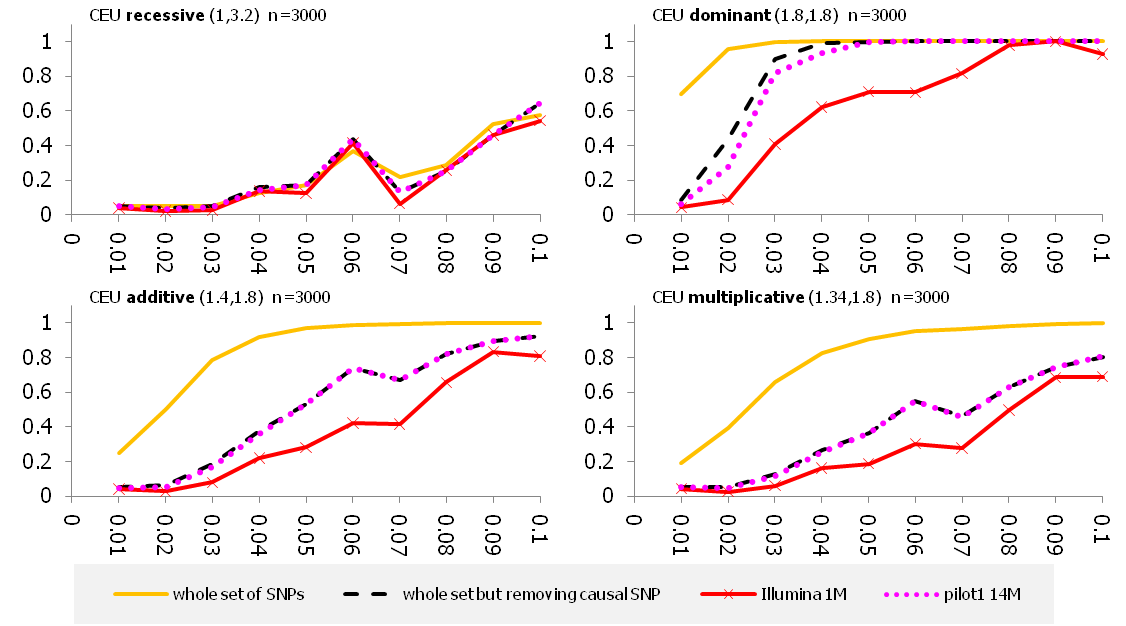

Supplement: Additional file 10 — Figure S8 - Power estimations for testing association of the low frequency allele from the CEU population with simulated sample size n = 3000 after imputation. The X axis represents allele frequency and the Y axis represents power. The solid line represents the power curve for the whole set of SNPs from 382 genes of the CEU population in the exon pilot, the dashed line represents the power curve for the set of all SNPs from 382 genes of the CEU population in the exon pilot except for the putative causal SNP, the dotted line represents the power curve for the low coverage Pilot 14 M dataset, and the solid line with the star represents the power curve for the Illumina 1 M. Values in parentheses are the heterozygous and homozygous relative risk, respectively. All putative causal SNPs in the low frequency region are presented. [file 1471-2164-12-322-S10.TIFF]
